# Embedded research to advance primary health care

**DOI:** 10.1136/bmjgh-2020-004684

**Published:** 2020-12-18

**Authors:** Soumya Swaminathan, Kabir Sheikh, Robert Marten, Martin Taylor, Manoj Jhalani, Ogochukwu Chukwujekwu, Luwei Pearson, Pascale Allotey, Jean Gough, Robert W Scherpbier, Anuradha Gupta, Marijke Wijnroks, Muhammad Ali Pate, Gaston Sorgho, Orin Levine, Felicity Goodyear-Smith, Thiagarajan Sundararaman, Hernan Montenegro, Suraya Dalil, Abdul Ghaffar

**Affiliations:** 1 Science Division, Organisation Mondiale de la Sante, Genève, Switzerland; 2 Alliance for Health Policy and Systems Research, World Health Organization, Geneva, Switzerland; 3 World Health Organization Regional Office for the Western Pacific, Manila, Philippines; 4 World Health Organization Regional Office for South-East Asia, New Delhi, Delhi, India; 5 UNICEF, New York, New York, USA; 6 IIGH, United Nations University International Institute for Global Health, Cheras, Kuala Lumpur, Malaysia; 7 UNICEF, Kathmandu, Nepal; 8 UNICEF, Geneva, Switzerland; 9 GAVI Alliance, Geneva, Genève, Switzerland; 10 The Global Fund to Fight AIDS, Tuberculosis and Malaria, Grand-Saconnex, Genève, Switzerland; 11 World Bank, Washington, DC, USA; 12 Bill & Melinda Gates Foundation, Seattle, Washington, USA; 13 General Practice, University of Auckland Faculty of Medical and Health Sciences, Auckland, New Zealand; 14 People's Health Movement, Mumbai, India; 15 World Health Organization, Geneva, Switzerland

**Keywords:** health policy, health systems

Realising renewed efforts to accelerate progress towards universal health coverage, manage health emergencies and create healthier populations requires integrated action on system-wide challenges. One of the best ways to do this is to focus on primary health care (PHC). Advancing PHC needs the support of a robust learning agenda—encompassing questions on how to improve both essential and emergency services, engage and empower communities and address the broader determinants of health through multisectoral action. Nowhere has the importance of learning been more compellingly exposed than during the ongoing COVID-19 pandemic, where the ability of countries to learn from experience has often determined the success or failure of their responses.

How research informs policy and action is variable, and in low- and middle-income countries, the contribution of research to decision-making is often limited. Traditionally, PHC research priorities are determined by funders or researchers based in high-income countries.^1^ This has constrained the utility of this research by overlooking important local context. Furthermore, traditional approaches to research are often static and not responsive.^2^ Embedding research to support policymaking is a demand-driven and contextually sensitive approach to redress this imbalance and create a learning approach to advance PHC.^3^

Embedded research is research carried out as an integrated and systematic part of decision-making and implementation.^4^ It involves a continuous role for policymakers, implementers and communities to collaborate and conduct research. It improves ownership, and consequently the utilisation of findings by its end users. The approach builds on the philosophies of research coproduction and the institutionalisation of research both endorsed and advocated for in the WHO Strategy on Health Policy and Systems Research (2012) and the WHO World Health Report on Research for Universal Health Coverage (2013). Since these reports, this approach has grown in the scale of its application and achieved demonstrable impact.

Embedded research has strengthened efforts to improve PHC—from helping develop guidelines and support packages for village health committees in India^5^ to the creation of action plans in Nigeria for improving immunisation services in underserved areas. For example, immunisation coverage in parts of Nigeria increased from 61% to 91%.^6^ The current COVID-19 pandemic also exemplifies how countries focused on learning can improve their responses. For instance, Thailand’s long-standing investments in PHC and in embedded research within the Ministry of Health have provided the basis for its response to COVID-19.^7^

Most importantly, embedded research is now embraced by its end users—health policymakers. Policymakers increasingly depend on support from researchers and communities to develop and implement policies to respond to the ongoing COVID-19 pandemic. In July 2020, one hundred and thirty policymakers and researchers from more than 20 countries in Asia and the Pacific came together to endorse the embedded research approach and highlight PHC research priorities with a focus on the COVID-19 response.^8^ Embedding research needs to become an integral part of the global push for PHC. Considering the ongoing COVID-19 pandemic, embedded PHC research should focus on scaling up and managing critical emergency services and continuing essential health services. In alignment with the 2018 PHC Astana Declaration, it is equally important to engage communities and address broader health determinants and multisectoral engagement.

As countries plan massive new COVID-19 vaccination efforts, embedded research will be crucial. Operationalising this agenda requires catalysing local researcher–policymaker collaborations. Mainstreaming learning into the PHC effort requires allocating human and financial resources for embedded research and greater efforts to build capacity for PHC research. External funders can also provide catalytic support for locally produced research as well as strengthen local capacities to undertake and use research. Investing resources in embedded research will contribute relevant, timely and contextual knowledge to improve and inform health policies—this is critical to advance PHC, manage COVID-19 and create a healthier post-COVID-19 future.
